# Phenolic Compound Profile and Antioxidant Capacity of Flax (*Linum usitatissimum* L.) Harvested at Different Growth Stages

**DOI:** 10.3390/molecules28041807

**Published:** 2023-02-14

**Authors:** Francesco Gai, Michał A. Janiak, Katarzyna Sulewska, Pier Giorgio Peiretti, Magdalena Karamać

**Affiliations:** 1 Institute of Sciences of Food Production, National Research Council, Largo Paolo Braccini 2, 10095 Grugliasco, Italy; 2 Department of Chemical and Physical Properties of Food, Institute of Animal Reproduction and Food Research, Polish Academy of Sciences, Tuwima 10, 10-748 Olsztyn, Poland

**Keywords:** linseed, flavonoids, *C*-glycosides, morphological stages, aerial parts of the plant, radical scavenging activity, emulsion system oxidation

## Abstract

The profile of phenolic compounds changes during the growth of a plant and this change affects its antioxidant potential. The aim of this research has been to find the growth stage of flax with the highest antioxidant capacity, and to determine the phenolic compounds responsible for such a capacity. Flax was harvested in six growth stages: from stem extension to mature seeds. The phenolic compounds were identified using LC–TOF–MS and quantified in an extract and in the fresh matter (FM) of each growth stage. The radical scavenging activity against ABTS^•+^ and DPPH^•^, the ferric-reducing antioxidant power (FRAP), and the antioxidant activity in the β-carotene-linoleic acid emulsion system were determined. Mono- and di-*C*-glycosyl flavones were found to be the most abundant phenolics of the aerial parts of flax, which also showed the highest content of isoorientin (210–538 µg/g FM). Coniferin, its derivative, and hydroxycinnamic acid derivatives were also detected. The plant was richer in flavone *C*-glycosides from stem extension to seed ripening (1105–1413 µg/g FM) than at the mature seed stage (557 µg/g FM). Most of the individual flavone *C*-glycoside contents in the extracts decreased when increasingly older plants were considered; however, the isoorientin content did not change significantly from the steam extension to the seed ripening stages. The antiradical activity against ABTS^•+^ and FRAP was higher for the aerial parts of the flax harvested at the flowering, brown capsule, and seed ripening stages, mainly due to the presence of flavone *C*-glycosides. The oxidation of β-carotene-linoleic acid emulsion was instead inhibited more effectively by the extracts from plants at the brown capsule and mature seed stages. Coniferin and its derivative were significantly involved in this activity. The extracts from the aerial parts of the flax harvested from flowering to seed ripening could be a valuable source of flavone *C*-glycosides for use as nutraceuticals and components of functional foods.

## 1. Introduction

Flax (*Linum usitatissimum* L.) is a plant that was domesticated thousands of years ago and is still cultivated today for its stems, which are sources of fiber that are used in textile production, and for the seeds, which contain an edible oil rich in *n*−3 fatty acids, mainly α-linolenic acid [1]. Nowadays, interest in other flax parts is growing, and this traditional dual-purpose crop has the potential to become a multi-purpose crop [1]. Flaxseed cake, the main by-product after oil pressing, is of particular interest, due to its high nutritionally valuable protein content [2], and the bioactive properties of peptides released from these proteins after their enzymatic hydrolysis [3,4], not only for feed animal producers, but also for human nutritionists and functional food producers. It contains mucilage, which is considered for food applications [5], and lignocellulose, which can be used in environmentally friendly polymeric composites [6]. Straw, a residue from both fiber and oil productions, is also a rich source of lignocellulose, which has the potential of being used as a bioplastic component and as biomass for bio-energy production [1]. However, in recent years, scientists have been paying more attention to flax bioactive compounds, especially phenolic compounds [7,8,9].

Flaxseeds are known to be a rich source of dietary lignans [10]. Of these phenolic compounds, secoisolariciresinol diglucoside (SDG) is the most abundant in flaxseed. Secoisolariciresinol and its derivatives, matairesinol, pinoresinol, lariciresinol, and isolariciresinol, have also been detected, but in low amounts [11]. SDG is metabolized, by intestinal bacteria, into mammalian phytoestrogens, i.e., enterolactone and enterodiol, which inhibit the growth of hormone-sensitive cancerous tumors [10]. In addition, lignans may prevent cardiovascular diseases, metabolic syndrome, and diabetes [12]. They have also shown high antioxidant activity [13,14]. Hydroxycinnamic acids, including the glucosides of *p*-coumaric, caffeic, and ferulic acids, as well as flavonoids, mainly herbacetin diglucoside, are other phenolic compounds of flaxseeds [7,15,16]. Garros et al. noted that the flax cultivar, weather conditions during plant growth, and crop location all affected the flaxseed phenolic content [7]. The degree of seed maturity is also an important factor that influences the phenolic profile [15]. SDG has been determined, in free form, at the early developmental stages, but, at the later developmental stages, SDG has been found in the ester-linked form and the contents of hydroxycinnamic acids and herbacetin diglucoside have increased. In other study, it has been reported that the seed maturity stage affected the antioxidant potential of flaxseeds, which has been found to be higher for flaxseeds at a mature stage [17].

The phenolic compounds of other flax organs have been studied less, and knowledge about them is limited; however, as early as 1971, Dubois and Marby identified mono- and di-*C*-glycosyl flavones (derivatives of apigenin and luteolin) in flax leaves and stems using nuclear magnetic resonance (NMR) [18]. The presence of isoorientin, isovitexin, and vitexin in the stems was later confirmed by means of ultra-performance liquid chromatography (UPLC) [19]. Tchoumtchoua et al. conducted a more thorough analysis using NMR and liquid chromatography-mass spectrometry (LC–MS) techniques in which they compared the phenolic profiles of leaves of winter and spring flax varieties, and they also detected, in addition to *C*-glycosyl flavones, coniferyl alcohol derivatives, although the latter was only in a small amount [9]. The effect of treating flax roots with chitosan oligosaccharides on the changes in the profile of secondary metabolites has recently been studied [20]. The authors reported the accumulation of (neo)lignans in roots, and luteolin *C*-glycosides and chlorogenic acid in shoots in response to stress. Pontarin et al. determined the metabolic profiles of flax leaves and found that the distribution of flavone *C*-glycosides was dependent on the age of the leaves; these phenolics were mainly accumulated in young leaves, and their content decreased with leaf development [21]. However, to the best of our knowledge, the phenolic profile of other organs or the aerial parts of flax have not been studied considering their age or lifecycle stage. Data on the bioactive potential of flax organs other than seeds are also lacking. Only in one study was the antioxidant potential of the green tissue of transgenic flax compared with a traditional cultivar [19]. On the other hand, flavonoid *C*-glycosides have well-documented biological activities, including antioxidant, anti-inflammatory, antimicrobial, anticancerous, antidiabetic, and hepatoprotective properties [22]. Therefore, the aim of our research has been to compare the phenolic profile and antioxidant capacity of flax harvested at different growth stages to establish the highest antioxidant capacity for future use in functional food applications, and to distinguish the phenolic compounds responsible for such bioactivity.

## 2. Results and Discussion

### 2.1. Extraction Yield and Total Phenolic Content

The lifecycle of flax, from sowing to maturity, depends on the overall environmental conditions, and is usually 90 to 125 days, with a vegetative period of 45 to 60 days, 15 to 25 days for the flowering period, and 30 to 40 days for the ripening period [23]. In our study, the aerial parts of flax were harvested 45, 51, 58, 65, 72, and 99 days after sowing, i.e., in the stem extension, visible bud, flowering, brown capsule, seed ripening, and mature seed stages, respectively. These plant materials were extracted using 80% (*v*/*v*) methanol. The extraction yield ranged from 10.2% (mature seed stage) to 20.4% (stem extension stage), and generally decreased as older plants were extracted (Table 1). A similar trend was noted in previous studies of ours, in which the aerial parts of other plants, including amaranth, false flax, soy, and sunflower were used [24,25,26,27].

The total phenolic contents (TPC) of the flax extracts are shown in Table 1. They ranged from 27.9 to 33.6 mg gallic acid equivalent (GAE)/g extract, and they did not differ significantly among the extracts obtained from plants at different growth stages (*p* ≥ 0.05). However, TPC, expressed on the basis of fresh matter (FM), changed significantly (*p* < 0.05) during the plant lifecycle, and was higher for flax harvested in the period from flowering to seed ripening (1.22–1.43 mg GAE/g FM) than at the beginning and at the end of the growth (0.88–1.06 mg GAE/g FM). To the best of our knowledge, the TPC of aerial parts of flax from field cultivation has not yet been analyzed, although the obtained values were in line with literature data for the aerial parts of amaranth (0.71–1.11 mg GAE/g FM) and sunflower (0.54–1.03 mg GAE/g FM), and were half the amount of those reported for false flax (1.46–3.10 mg GAE/g FM) at different growth stages [24,25,26]. The higher TPC of flax at the flowering and seed development than at the stem extension and visible bud stages was also consistent with previous studies on sunflower and amaranth [24,25], and, according to the optimal defense theory (ODT) [28], indicated that flowers and seeds were defended more than the other organs. The assumption of ODT is that the allocation of secondary metabolites to the plant is optimized and the plant therefore incurs the lowest possible costs of defending the tissues and organs against biotic and abiotic stress. The content of the phytochemicals is higher in those organs that are the most useful for plant growth and reproduction. However, although this was not observed in our study, leaves sometimes also meet this criterion [29,30].

The values of TPC for the flax extracts were quite high, compared to those reported in the literature for the aerial parts of several herbs (28.07, 32.91, and 51.04 g GAE/kg extract for coriander, tarragon, and lovage, respectively), which are considered to be rich sources of phenolic compounds [31].

### 2.2. Phenolic Compound Profile

**Figure 1:**
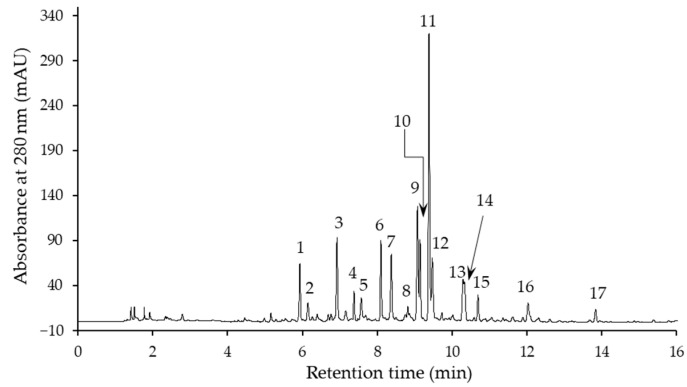
Chromatogram of a flax extract obtained by means of high-performance liquid chromatography with a diode-array detector (HPLC–DAD).

Seventeen phenolic compounds were determined in the flax extracts by means of liquid chromatography, together with time-of-flight mass spectrometry (LC–TOF–MS) and high-performance liquid chromatography with a diode-array detector (HPLC–DAD). An example of an HPLC–DAD profile of the phenolic compounds of an extract is shown in Figure 1. The peaks on the chromatogram correspond to compounds **1**–**17**, whose UV-DAD spectrum maxima (λ_max_) and the parent and characteristic MS^2^ ions are listed in Table 2. These data were used to identify compounds. The most polar compound (**1**), with λ_max_ of the UV spectrum at 259 nm, an [M−H]^−^ ion at *m/z* 341, and an MS^2^ base fragment ion of [M−H−162]^−^ at *m*/*z* 179, was found to be coniferin. The presence of coniferin in flax leaves, and in immature seeds and roots, had previously been reported [9,15,20,32]. Compounds **2** and **3** showed a similar [M−H]^−^ ion and an MS^2^ base fragment ion to compound **1**, but a different UV spectrum (characteristic of caffeic acid). They were tentatively identified as caffeic acid hexosides. Compound **5** was instead identified as *p*-coumaric acid ethyl ester on the basis of its λ_max_ of UV spectrum and a parent ion at *m*/*z* 191.

As many as six compounds (**4**, **6**–**10**) were recognized as di-*C*-glycosyl flavones. Fragment ions at *m*/*z* 353 (Ag + 83) and at *m*/*z* 383 (Ag + 113) were found to be characteristic of apigenin *C*-glycosides, and those at *m*/*z* 369 (Ag + 83) and at *m*/*z* 399 (Ag + 113) were identified as diagnostic ions for luteolin di-*C*-glycosides [33,34]. In our study, the detection of these ions (Table 2) enabled compounds **6**, **8**–**10** to be included in the apigenin di-*C*-glycosides, and compounds **4** and **7** to be included in the luteolin di-*C*-glycosides. Additional evidence for such a classification was provided by the UV spectra, with different λ_max_ for each group (Table 2). The literature has reported λ_max_ at 270–272 and 333–338 nm for apigenin di-*C*-glycosides, and a shift in the second maximum toward a longer wavelength of 343–349 nm for luteolin di-*C*-glycosides [34,35,36]. Further identification was based on preferential cleavages of the sugar moieties and relative intensity of the [M−H−120]^−^, [M−H−90]^−^, and [M−H−60]^−^ fragment ions [33,34]. A lack of an [M−H−60]^−^ fragment ion and the simultaneous high intensity of the produced [M−H−120]^−^ ion suggested that the compound had the structure of flavone with 6,8-di-*C*-symmetric glycosides. Accordingly, compound **4,** which showed an [M−H]^−^ ion at *m*/*z* 609, an [M−H−120]^−^ base fragment ion at *m*/*z* 489, an [M−H−90]^−^ less-intense ion at *m*/*z* 519, and an [M−H−18]− ion at *m*/*z* 591, was tentatively identified as luteolin 6,8-di-*C*-hexoside. Since lucenin-2 (luteolin 6,8-di-*C*-glucoside) has already been detected in flax leaves, stems, and shoots [9,18,20,21], we assumed that this was compound **4**. In a similar manner, an [M−H]^−^ ion at *m*/*z* 593, an [M−H−120]^−^ MS^2^ base ion at *m*/*z* 473, a less-intense [M−H−90]^−^ ion at *m*/*z* 503, and an [M−H−18]^−^ ion at *m*/*z* 575, led to the identification of compounds **6** and **8** as being apigenin 6,8-di-*C*-hexosides. The structure of the former was then confirmed by comparing chromatographic and spectral data with the standard of vicenin-2 (apigenin 6,8-di-*C*-β-d-glucoside). Vicenin-2 had previously been found in flax leaves, stems, and shoots [9,18,20,21]. Compound **10** showed an [M−H]^−^ ion at *m*/*z* 563, and [M−H−120]^−^, [M−H−90]^−^, and [M−H−60]^−^ ions, produced from cleavages of the sugar moieties, at *m*/*z* 443, *m*/*z* 473, and *m*/*z* 503, respectively. An [M−H−18]^−^ ion was also found at *m*/*z* 545. This compound was recognized as schaftoside (apigenin 8-*C*-α-l-arabinoside 6-*C*-β-d-glucoside), and its identification was confirmed through a comparison with an analyzed commercial standard. The parent and MS^2^ ions of compound **9** were similar, although the intensity of the [M−H−60]^−^ ion at *m/z* 503 was higher than that of schaftoside, and it was therefore tentatively identified as apigenin 8-*C*-hexoside 6-*C*-pentoside. Among the apigenin 6,8-di-*C*-glycosides with pentose and hexose moieties, schaftoside and its isomer were detected in the flax leaves and shoots [9,20,21], while vicenin-1 (apigenin-6-*C*-xyloside-8-*C*-glucoside) was identified in the leaves, stem, and shoots [9,18,20,21]. Moreover, three apigenin *C*-hexoside *C*-pentoside isomers were tentatively identified in the flaxseed cake [37]. Compound **7,** which showed a parent ion at *m/z* 579 and a similar fragmentation pathway, with neutral losses of 120, 90, and 60 Da from an [M−H]^−^ ion, was classified as luteolin 6,8-*C*-hexoside-*C*-pentoside. Carlinoside, the carlinoside isomer, and lucenin-1, which belong to luteolin 6,8-di-*C*-asymmetric glycosides (with glucose and arabinose moieties), had previously been found in different organs of flax [9,18,20,21].

Compounds **11**, **12**, **13,** and **15** were identified as isoorientin, orientin, vitexin, and isovitexin, respectively, on the basis of their λ_max_ of the UV spectra, the [M−H]^−^ ions at *m/z* 447 (for luteolin *C*-glycosides), and *m*/*z* 431 (for apigenin *C*-glycosides), as well as the fragmentation pathway (Table 2), which was in line with the literature findings [33,34]. These mono-*C*-glucosyl derivatives of apigenin and luteolin have been found in flax organs (leaves, stems, and shoots) [9,18,19,20,21], as well as in flax by-products after oil pressing, i.e., seed cake and straw [8,37].

Tchoumtchoua et al. [9] detected an [M−H]^−^ ion at *m/z* 565, as well as its fragment ions at *m*/*z* 339 and at *m*/*z* 327, when analyzing a flax leaf extract by means of LC–TOF–MS and found that they were from a dehydrodiconiferyl alcohol-4-*O*-glucoside (DCG) adduct with COOH^−^. The identification was confirmed by means of the standard. In our study, the same parent and MS^2^ ions were determined for compound **14**. Additionally, MS^2^ ions were detected at *m*/*z* 519 ([M_DCG_−H]^−^), and at *m*/*z* 357 ([M_DCG_−H−162]^−^) (Table 2). Therefore, compound **14** was identified as dehydrodiconiferyl alcohol-4-*O*-glucoside. Compound **16** was tentatively identified as a caffeic acid derivative, on the basis of its UV spectrum, as its shape and λ_max_ were typical of caffeic acid. An [M−H]^−^ ion at *m*/*z* 685 suggested that compound **17** could be an SDG. On the other hand, the fragmentation ions, shown in Table 2, do not agree with those described in the literature for this compound (which indicates the loss of a glucose moiety) [38,39,40]. SDG had previously been determined, in free form, in immature flaxseeds and sprouts [15,32,41]. However, its detection in the aerial parts of young flax would be unexpected, because pinoresinol-lariciresinol reductase (PLR), which catalyzes the conversion of pinoresinol into secoisolariciresinol in the early steps of lignan biosynthesis, shows an opposite enantioselectivity in leaves and stems to seeds [32,42]. Only the enantiomer of secoisolariciresinol synthetized in the seeds is glycosylated to SDG and accumulated in this form. The enantiomer synthesized in leaves and stems is a precursor of yatein. In short, the full structure of compound **17** remains unknown.

The quantitative phenolic profiles of the extracts of flax harvested at different growth stages are shown in Table 3. The main phenolic of the extracts was isoorientin, with a content of 7.90–14.3 mg/g. The contents of the second mono-*C*-glucosyl luteolin (orientin), and both di-*C*-glycosides (lucenin-2 and luteolin 6,8-*C*-hexoside-*C*-pentoside), were also quite high. They ranged from 2.04 to 4.80, 0.880 to 1.46, and 3.08 to 3.77 mg/g extract, respectively. Among the apigenin derivatives, the di-*C*-glycosides were more abundant than the mono-*C*-glycosides. The contents of most of the former were 2–3 times higher than those of vitexin. Isovitexin was present in small amounts, that is, below 1 mg/g extract, as obtained from flax harvested at most growth stages. The total content of flavone *C*-glycosides ranged from 21.0 to 36.8 mg/g extract. Considering the differences in the growth phases, the contents of the individual compounds of this group, including vicenin-2, apigenin 6,8-di-*C*-hexoside, apigenin 8-*C*-hexoside 6-*C*-pentoside, orientin, and vitexin, decreased in the extracts prepared from increasingly older plants. However, the contents of the most abundant compound, that is, isoorientin, did not differ significantly (*p* ≥ 0.05) between extracts of the flax at the stem extension, flowering, and brown capsule stages. This resulted in the same differences between the growth stages, in terms of the total flavone *C*-glycoside content. The amounts of the other phenolic compounds in the extracts were relatively low (mostly below 1 mg/g extract), but interestingly, the contents of coniferin, DCG, and an unidentified compound (**17**) tended to increase in the extracts from increasingly older plants.

The extracts of the aerial parts of the flax were generally rich sources of flavone *C*-glycosides. Glycosyl flavonoids, with their *C*–*C* bond between the aglycone backbone and a sugar moiety in the structure, are less common in plants used traditionally for food than those with a sugar moiety linked to the aglycone by an *O*-glycosidic bond [43]. The type of bond between the sugar moiety and the aglycone determines the properties of the compounds [22,44]. *C*-Glycosylated flavonoids are more stable, which may be important for the processing plants that contain these compounds. In addition, flavonoids with both types of glycosidic bonds differ in their biological activities [44]. Flavone *C*-glycosides, as mentioned above, are known for their broad bioactivity, especially antioxidant, anticancerous, and antidiabetic properties [22]. Czemplik et al. reported that flavone *C*-glycosides derived from flax straw induced growth inhibition and apoptosis in the cells of the human breast adenocarcinoma line (MCF-7) [8]. In this context, the extracts of the aerial parts of flax, especially when harvested at the stem extension, flowering, and brown capsule stages, can be considered as valuable ingredients to design functional foods and nutraceuticals.

The content of the phenolic compounds in the fresh matter of the aerial parts of flax is shown in Table 4. The proportions of the individual phenolic contents between the growth stages were slightly different from those in the extracts. The contents of lucenin-2, luteolin 6,8-*C*-hexoside-*C*-pentoside, isoorientin, and orientin did not change significantly (*p* ≥ 0.05) from the stem extension to seed ripening stages, and they were only lower at the mature seed stage. Decreases in the contents of all the apigenin di-*C*-glycosides began earlier, at the seed ripening stage. Other compounds (coniferin, schaftoside, vitexin, and isovitexin, as well as an unidentified compound) were the most abundant at the flowering, brown capsule, and/or seed ripening stages. A significantly lower content (*p* < 0.05) was found at the mature seed stage than at the other growth stages, for all the individual phenolics (with the exception of DCG), for the total phenolics, and total flavone *C*-glycosides. This may be due to the relatively high proportion of seeds in the aerial parts of flax at the mature seed state. Flavones were not identified among the flaxseed phenolics. SDG, which is the dominant phenolic, and herbacetin diglucoside (HDG), *p*-coumaric acid glucoside, caffeic acid glucoside, and ferulic acid glucoside have been detected in seeds [7,15,45]. However, these compounds are not found in free form in mature seeds [15]. They are accumulated as lignan macromolecules, where SDG and HDG are ester-linked to 3-hydroxy-3-methylglutaric acid, and hydroxycinnamic acid glucosides are directly bound to SDG [7,16]. Therefore, the quantitative determination of seed phenolics by means of HPLC is only possible after cleavage of the ester bonds of the lignan macromolecule [38,45,46]. For these reasons, the underestimation of the phenolics in the macromolecules of seeds, which constitute a large percentage of the mass of the aerial part of flax, may explain the lower content of phenolics at the mature seed stage.

In a previous study, isoorientin was found to be the predominant compound in flax stems, and its content was approximately twice that of isovitexin and vitexin [19]. These data are consistent with the results of our research on the aerial parts of flax, probably because the stems constituted a large mass in most of the analyzed growth states. However, Tchoumtchoua et al. found orientin to be the main phenolic in the leaves of spring flax varieties. Its content was much lower (12 times) in winter varieties, which were dominated by swertiajaponin, vicenin-1, and vicenin-2 [9]. It was found that the content of flavone *C*-glycosides depended on the age of the leaves, and flavone di-*C*-glycosides were mainly accumulated in young leaves [21]. Such an observation would seem to suggest a greater contribution of di-*C*-glycosides at the earliest growth stage (stem extension), but no confirmation has been found. It is likely that the phenolic profile of other organs covers these age-dependent differences in leaves.

### 2.3. Antioxidant Capacity

The antioxidant capacity of the aerial parts of flax harvested at different growth stages was determined by performing several assays to estimate the activity of the antioxidants on the basis of their various modes of action. The results of the Trolox equivalent antioxidant capacity (TEAC), DPPH^•^ scavenging activity, ferric-reducing antioxidant power (FRAP), and inhibition of β-carotene-linoleic acid emulsion oxidation are shown in Table 5.

**Figure 2:**
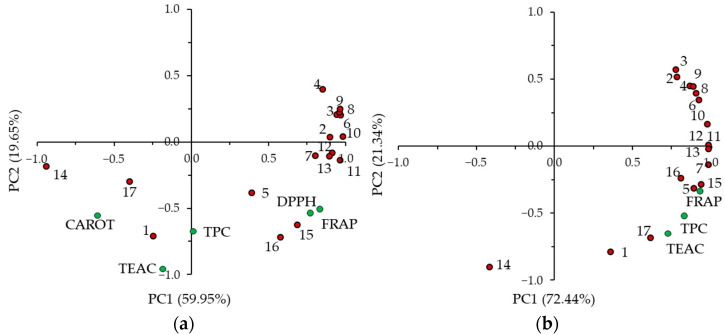
Principal component analysis (PCA) plots with distribution of the variables of the flax extracts (**a**) and of the fresh matter of flax (**b**); 1–17, contents of the individual phenolic compounds (see Table 2); CAROT, inhibition of β-carotene-linoleic acid emulsion oxidation; DPPH, DPPH^•^ scavenging activity; FRAP, ferric-reducing antioxidant power; TEAC, Trolox equivalent antioxidant capacity; TPC, total phenolic content.

The TEAC of the extracts was in the 0.150–0.226 mmol TE/g range and did not differ significantly (*p* ≥ 0.05) in the extracts obtained from plants at different growth states. The differences for FRAP, expressed on the basis of the extracts, were also slight (0.669–0.783 mmol Fe^2+^/g extract). The TEAC of the flax FM was higher at the seed ripening, flowering, and brown capsule stages, with values that did not differ significantly (*p* ≥ 0.05) from each other. Similarly, the highest FRAP of the flax FM was found for flax plants at the seed ripening, flowering, and brown capsule stages. The TEAC of the extracts and the TEAC and FRAP of the flax FM were closely correlated with TPC, as indicated by a principal component analysis (PCA) (Figure 2). The mentioned variables are clustered on the plots. This is not surprising, considering that the mechanism of action of the antioxidants in all three assays was via a single electron transfer reaction [47]. In a previous study of ours, close relationships between the TPC, FRAP, and TEAC of the aerial parts of amaranth and false flax were found, at various growth stages, by means of a PCA [25,26]. Significant Pearson’s correlations, with high correlation coefficients (0.916–0.977), were noted for the TPC, FRAP, and TEAC of the aerial parts of sunflowers collected during their growth cycle [24].

The flax extracts were analyzed in a DPPH assay, and once again, the differences in the growth stages were slight (Table 5). The highest DPPH^•^ scavenging activity was observed for the extract from the plant at the brown capsule stage (EC_50_ of 0.152 mg/mL), although the value did not differ significantly (*p* ≥ 0.05) from those of the extracts at the stem extension, flowering, and seed ripening stages. The results of the DPPH assay correlated well with those of FRAP (Figure 2a). In contrast to FRAP and the ABTS^•+^ and DPPH^•^ scavenging activity, the inhibition of β-carotene-linoleic acid emulsion oxidation was higher for the extracts from flax harvested at the mature seed and brown capsule stages. A PCA confirmed the weak relationship between the antioxidant activity measured in the β-carotene-linoleic acid system and in the other assays, especially for FRAP and DPPH (Figure 2a). This phenomenon could be due to the different action mechanism in the β-carotene-linoleic acid assay, in which the radicals that form during lipid peroxidation are quenched by a hydrogen atom transfer reaction [47]. Different phenolic compounds probably play significant roles in both types of assays. In addition, the polarity of the measurement systems could be important, as well as the different activities of the compounds under polar conditions (FRAP, ABTS, and DPPH assays) and in the lipid emulsion [26].

The PCA was employed to assess which phenolic compounds were responsible for the antioxidant capacity of the aerial parts of flax. The first two principal components (PC1 and PC2) explained 79.60% (PC1–59.95% and PC2–19.65%) and 93.78% (PC1–72.22% and PC–21.34%) of the total variability of the extracts and flax FM, respectively (Figure 2). Considering the data set of the extract, it clearly emerged that there was discrimination along PC1 between compounds **1**, **14,** and **17**, i.e., coniferin, its derivative, and an unidentified compound, and the remaining compounds, which have been classified as flavone *C*-glycosides and caffeic acid derivatives (Figure 2a). The first group of compounds was clustered together with the antioxidant activity measured in the β-carotene-linoleic acid system on the plot, and the second one together with FRAP and the DPPH. This confirmed our supposition mentioned above that the various compounds were determinants of antioxidant activity in assays under polar and lipid conditions. The clustering of the variables was not so evident for FM (Figure 2b). Most of the phenolic compounds, and the TPC, TEAC, and FRAP did not discriminate along PC1. Nevertheless, the distribution of the variables showed the contribution of most of the phenolic compounds in the antioxidant activity of the aerial parts of flax as determined by TEAC and FRAP.

## 3. Materials and Methods

### 3.1. Plant Material and Cultivation Conditions

Brown variety flaxseeds were provided by Ornitalia Product Service (Colleredo di Monte Albano, Italy). They were sown in 3 × 12 m^2^ experimental plots in the Western Po Valley (44°41′ N, 7°11′ E), Italy. Cultivation was carried out without fertilization or irrigation. The aerial parts of the flax were harvested from July to September, on rainless days, on randomly selected 2 m^2^ plots at six morphological stages, that is, from stem extension to the mature seed stage (Table 1). Three replicates were collected for each harvest period. The morphological stages were assessed on a sample of approximately 50 stems and classified according to a 12-stage grading system developed by the Flax Council of Canada [23]. Whole plants, cut 1–2 cm above ground level, were immediately transported to the laboratory of the NRC Institute of Sciences of Food Production in Grugliasco, Italy, where they were frozen and freeze-dried using a 5 Pascal device (Trezzano sul Naviglio, Milan, Italy). After drying, the material was ground to particles by passing it through a 1 mm screen, and it was then tightly closed in vessels and stored, at −20 °C, for future analysis.

### 3.2. Chemicals

Folin-Ciocalteau’s phenol reagent (FCR), 2,2′-diphenyl-1-picrylhydrazyl (DPPH) radicals, gallic acid, 2,2′-azinobis-(3-ethylbenzothiazoline-6-sulfonic acid) diammonium salt (ABTS), 6-hydroxy-2,5,7,8-tetramethyl-chroman-2-carboxylic acid (Trolox), 2,4,6-tri(2-pyridyl)-*s*-triazine (TPTZ), β-carotene, linoleic acid, Tween 40, HPLC standards, including caffeic acid, *p*-coumaric acid, orientin (luteolin 8-*C*-β-d-glucoside), isoorientin (luteolin 6-*C*-β-d-glucoside), vitexin (apigenin 8-*C*-glucoside), isovitexin (apigenin 6-*C*-glucoside), vicenin-2 (apigenin 6,8-di-*C*-β-d-glucoside), and schaftoside (apigenin 8-*C*-α-l-arabinoside 6-*C*-β-d-glucoside) were obtained from Sigma-Aldrich (St. Louis, MO, USA). Sodium persulfate, ferrous chloride, and the solvents were provided by Avantor Performance Materials (Gliwice, Poland).

### 3.3. Preparation of the Extracts

The phenolic compounds were extracted from freeze-dried and ground flax using a methanol:water (5:1, *v*/*v*) mixture. The extraction was carried out at a 1:10 (*v*/*w*) plant material-to-solvent ratio in closed glass vessels that were shaken in an SW22 water-bath (Julabo, Seelbach, Germany) at 65 °C [48]. A portion of 5 g of the freeze-dried and powdered flax was used for each extraction. Each sample was extracted three times for 15 min. The organic solvent was evaporated from combined filtrates, using a Rotavapor R-200 (Büchi Labortechnik, Flawil, Switzerland), and the remaining aqueous residue was freeze-dried (Lyph Lock 6 freeze-dry system, Labconco, Kansas City, MO, USA). The extraction yield (%) was calculated from the mass balance.

### 3.4. Determination of the Total Phenolic Content

The TPC of the flax was determined by means of an FCR assay, according to the procedure described in detail in a previous publication [27]. The absorbance was measured at 725 nm, by means of a DU-7500 spectrophotometer (Beckman Instruments, Brea, CA, USA). The results were expressed as mg of gallic acid equivalent (GAE) per g of extract or per g of plant FM.

### 3.5. Phenolic Compound Profile Analysis

An Eksigent microLC 200 system, with a TripleTOF 5600+ mass spectrometer (AB Sciex, Framingham, MA, USA), was used to detect the phenolic compounds of the flax. Flax extracts were injected into an Eksigent Halo C18 column (0.5 × 50 mm, 2.7 μm; AB Sciex) and separations were performed in a linear gradient system of 0.1% (*v*/*v*) formic acid in water and 0.1% (*v*/*v*) formic acid in acetonitrile (with an increase in the proportion of the second solution, from 1 to 90%, over 3 min) as the mobile phase [24]. An electrospray ionization source, operating in negative mode (ion spray voltage 4.5 kV), was used. The flow rate of the nebulizer and curtain gases was 30 L/min, and that of the heater gas was 35 L/min. The other MS conditions were as follows: turbo spray temperature, 350 °C; declustering potential (DP) and collision energy (CE) for full-scan MS, 90 V and 20 eV, respectively; and, for MS^2^ mode, 80 V and 30 eV, respectively. The TOF–MS was operated over the 100–1200 mass range.

Quantification of the phenolic compounds of the flax extracts was carried out using an HPLC system (Shimadzu, Kyoto, Japan) with SPD-M30A DAD. An aliquot of 1.5 µL of extract solution, in 80% (*v*/*v*) methanol (20 mg/mL), was injected into a Kinetex C18 column (4.6 × 150 mm, 2.6 μm, Phenomenex, Torrance, CA, USA). A gradient system of the mobile phase, consisting of acetonitrile-water-trifluoroacetic acid, 5:95:0.1, *v*/*v*/*v* (solvent A), and acetonitrile-trifluoroacetic acid, 100:0.1, *v*/*v* (solvent B), was used [49]. It was pumped at a flow rate of 1 mL/min into a linear gradient system of 0–30% B over a period of 15 min. The DAD was set at a wavelength range of 200 to 400 nm. Quantification of the compounds was based on the calibration curves of caffeic acid, *p*-coumaric acid, orientin, isoorientin, vitexin, isovitexin, vicenin-2, and schaftoside.

### 3.6. Trolox Equivalent Antioxidant Capacity Determination

The antiradical activity against ABTS^•+^ was determined as the TEAC. ABTS^•+^ was generated and diluted according to the method of Re et al. [50]. Before the spectrophotometric measurement, 2 mL of the ABTS^•+^ solution and 20 µL of a flax extract solution (3 mg/mL) were mixed, and the mixture was warmed at 37 °C in the dark (TH-24 block heater, Meditherm, Warsaw, Poland) for 6 min. The spectrophotometer (Beckman DU-7500) was set at 734 nm. The results were expressed as mmol Trolox equivalent (TE) per g of extract or μmol TE per g of plant FM.

### 3.7. Determination of the Ferric-Reducing Antioxidant Power

The FRAP of the flax was assayed by means of the Benzie and Strain method [51]. Briefly, the aqueous extract solutions were prepared at a concentration of 1 mg/mL. The FRAP reagent was obtained by vortexing 10 mM TPTZ in a 40 mM HCl, 300 mM acetate buffer at pH 3.6 and 20 mM FeCl_3_ in a ratio of 1:10:1 (*v*/*v*/*v*). The aliquots of the FRAP reagent (2.25 mL) were warmed at 37 °C (TH-24 block heater). A 75 μL aliquot of the extract solutions and 0.225 mL of water were then added. The absorbance was measured at 593 nm (Beckman DU-7500 spectrophotometer) after 30 min of incubation. The FRAP results were expressed as mmol Fe^2+^ equivalent per g of extract or μmol Fe^2+^ equivalent per g of plant FM.

### 3.8. Determination of the DPPH Radical Scavenging Activity

The DPPH radical scavenging activity of the flax extracts was determined according to the method described by Brand-Williams et al. [52]. Briefly, 100 µL of an extract solution in methanol (concentration range from 1.2 to 6.0 mg/mL) was vortexed with 2 mL of methanol, and 0.25 mL of a DPPH^•^ solution in methanol (1 mM) was then added. The mixture was left to stand in the dark at an ambient temperature for 20 min, and absorbance was then measured using a Beckman DU-7500 spectrophotometer set at 517 nm. The absorbance values were plotted as a function of the extract concentration in the reaction mixture. The half-maximal effective concentration (EC_50_), i.e., the concentration of extract necessary to scavenge 50% of the initial DPPH^•^, was read from the plot.

### 3.9. β-Carotene-Linoleic Acid Emulsion Oxidation

A β-carotene-linoleic acid model system was used to determine the ability of the flax extracts to inhibit emulsion oxidation. An aqueous emulsion of linoleic acid and β-carotene was prepared, with Tween 40 as the emulsifier, as previously described [53]. The oxidation of emulsion was carried out in a 96-well plate at 42 °C. An aliquot of 250 µL of emulsion was pipetted into the well, and 20 µL of extract solution (1 mg/mL) was added. Methanol was used instead of the extract solution in the control sample. The plate was incubated in an Infinite M1000 microplate reader (Tecan, Männedorf, Switzerland). The absorbance was recorded at 470 nm for 180 min at 15 min intervals. The results were expressed as the percentage of non-oxidized β-carotene.

### 3.10. Statistical Analysis

Three samples of the aerial parts of flax were collected for each growth stage, and an extract was prepared separately from each sample. The chemical determinations were carried out at least in triplicate. The obtained results were reported as means with standard deviations. An analysis of variance (ANOVA) and Fisher’s least significant difference (LSD) test were performed to evaluate the significance of the differences in the mean values (*p* < 0.05). GraphPad Prism software (GraphPad Software, San Diego, CA, USA) was used for all the statistical calculations. A principal component analysis (PCA) was performed, using Statistica 13.1 software (StatSoft Corp., Kraków, Polska), to describe the variations in the TPC values, in the individual phenolic contents, and in the values of the antioxidant assays.

## 4. Conclusions

Flavone mono- and di-*C*-glycosides were found to be the most numerous compounds among the 17 phenolic compounds identified in the aerial parts of flax. They were also the most abundant phenolics. In addition, the derivatives of hydroxycinnamic acids, coniferin, and its derivative were also found. The profiles of the phenolic compounds in the aerial parts of the flax and their extracts were dependent on the plant growth stage. The flax from the stem extension to seed ripening stage was richer in total phenolic and total flavone *C*-glycosides than flax at the mature seed stage. Most of the content of the individual flavone *C*-glycosides decreased for the extracts prepared from increasingly older plants. The antioxidant capacity of the aerial parts of the flax was affected by both the plant growth stage and the mode of action of the assay used to measure the antioxidant activity. Flax harvested at the flowering, brown capsule, and seed ripening stages showed a high antioxidant capacity when evaluated as FRAP and TEAC. Flavone *C*-glycosides were the compounds that contributed the most to the antioxidant activity, as determined by these assays. Under a lipid condition, during the oxidation of β-carotene-linoleic acid emulsion, coniferin and its derivative were involved to a great extent in the antioxidant activity, which was higher for the extracts of flax harvested at the brown capsule and mature seed stages.

Since the aerial parts of flax are a rich source of flavone *C*-glycosides, a less common form of flavonoids than *O*-glyosides in edible plants, but which, and at the same time, have desirable properties, it seems that extracts from young flax could be considered in the design of functional foods.

## Figures and Tables

**Table 1:** Extraction yield and total phenolic content of the extracts and fresh matter (FM) of flax harvested at different growth stages.

Growth Stage	Days after Sowing	Extraction Yield (%)	Total Phenolic Content	
			mg GAE/g Extract	mg GAE/g FM
Stem extension	45	20.4 ± 0.8 ^a^	33.6 ± 2.9 ^a^	1.06 ± 0.17 ^b,c^
Visible bud	51	17.8 ± 1.2 ^b^	27.9 ± 4.8 ^a^	1.01 ± 0.13 ^b,c^
Flowering	58	16.9 ± 1.3 ^b,c^	29.2 ± 1.7 ^a^	1.33 ± 0.20 ^a^
Brown capsule	65	14.4 ± 0.1 ^d^	33.4 ± 2.1 ^a^	1.22 ± 0.09 ^a,b^
Seed ripening	72	16.0 ± 0.7 ^c^	32.8 ± 2.6 ^a^	1.43 ± 0.12 ^a^
Mature seed	99	10.2 ± 0.4 ^e^	33.1 ± 2.7 ^a^	0.88 ± 0.05 ^c^

GAE, gallic acid equivalent. Means with different letters in the same column are significantly different (*p* < 0.05).

**Table 2:** Absorption maxima (λ_max_) of the UV spectra from the HPLC–DAD analysis, and the parent and fragmentation ions from LC–TOF–MS analysis of the phenolic compounds of the flax extracts.

Compound No. ^1^	λ_max_ (nm)	[M−H]^−^ (*m*/*z*)	MS^2^ Ions (*m*/*z*)	Identified Compound
1	259	341	179, 161	Coniferin (coniferyl alcohol β-d-glucoside)
2	298 sh, 329	341	179, 161, 131	Caffeic acid hexoside I
3	299 sh, 326	341	179, 161	Caffeic acid hexoside II
4	256 sh, 271, 347	609	591, 519, 489, 399, 369	Lucenin-2 (luteolin 6,8-di-*C*-glucoside)
5	286, 314	191		*p*-Coumaric acid ethyl ester
6 ^2^	271, 336	593	575, 503, 473, 383, 353	Vicenin-2 (apigenin 6,8-di-*C*-β-d-glucoside)
7	256 sh, 270, 348	579	561, 519, 489, 459, 399, 369	Luteolin 6,8-*C*-hexoside-*C*-pentoside
8	271, 333	593	575, 503, 473, 383, 353	Apigenin 6,8-di-*C*-hexoside
9	271, 335	563	545, 503, 473, 443, 383, 353	Apigenin 8-*C*-hexoside 6-*C*-pentoside
10 ^2^	271, 336	563	545, 503, 473, 443, 383, 353	Schaftoside (apigenin 8-*C*-α-l-arabinoside 6-*C*-β-d-glucoside)
11 ^2^	256 sh, 269, 349	447	429, 357, 327, 297	Isoorientin (luteolin 6-*C*-β-d-glucoside)
12 ^2^	257 sh, 270, 349	447	429, 357, 327, 297	Orientin (luteolin 8-*C*-β-d-glucoside)
13 ^2^	269, 337	431	341, 323, 311, 283, 269	Vitexin (apigenin 8-*C*-glucoside)
14	272	565	519, 357, 339, 327	Dehydrodiconiferyl alcohol-4-*O*-glucoside (DCG)
15 ^2^	270, 338	431	341, 323, 311, 283, 269	Isovitexin (apigenin 6-*C*-glucoside)
16	299 sh, 330	401	222	Caffeic acid derivative
17	278	685	667, 521, 363, 303	Unknown

^1^ The compound numbers correspond to the peak numbers shown in Figure 1. ^2^ Compounds identified on the basis of the standards. Sh, shoulder.

**Table 3:** Contents of the individual phenolic compounds in the extracts of flax harvested at different growth stages (mg/g extract).

Compound	Stem Extension	Visible Bud	Flowering	Brown Capsule	Seed Ripening	Mature Seed
Coniferin ^1^	0.642 ± 0.028 ^c^	0.598 ± 0.086 ^c^	0.555 ± 0.023 ^c^	1.07 ± 0.16 ^b^	1.71 ± 0.27 ^a^	0.719 ± 0.118 ^b,c^
Caffeic acid hexoside I ^1^	0.628 ± 0.045 ^a^	0.341 ± 0.039 ^b,c^	0.397 ± 0.037 ^b^	0.439 ± 0.022 ^b^	0.291 ± 0.037 ^c^	0.280 ± 0.044 ^c^
Caffeic acid hexoside II ^1^	2.39 ± 0.18 ^a^	1.37 ± 0.10 ^b,c^	1.43 ± 0.16 ^b^	1.29 ± 0.06 ^b,c^	0.991 ± 0.161 ^c^	0.421 ± 0.121 ^d^
Lucenin-2 ^2^	1.46 ± 0.18 ^a^	1.23 ± 0.06 ^a,b^	1.05 ± 0.13 ^b,c^	1.14 ± 0.05 ^a,b,c^	0.880 ± 0.073 ^c^	0.907 ± 0.149 ^c^
*p*-Coumaric acid ethyl ester ^3^	0.224 ± 0.015 ^b^	0.232 ± 0.012 ^b^	0.233 ± 0.011 ^a,b^	0.261 ± 0.006 ^a^	0.216 ± 0.003 ^b^	0.223 ± 0.009 ^b^
Vicenin-2	3.43 ± 0.22 ^a^	2.78 ± 0.21 ^b^	2.89 ± 0.12 ^b^	2.87 ± 0.19 ^b^	2.05 ± 0.13 ^c^	1.82 ± 0.11 ^c^
Luteolin 6,8-*C*-hexoside-*C*-pentoside ^2^	3.77 ± 0.30 ^a^	3.21 ± 0.21 ^a,b^	3.19 ± 0.26 ^ab^	3.55 ± 0.14 ^a,b^	3.08 ± 0.16 ^b^	3.17 ± 0.26 ^a,b^
Apigenin 6,8-di-*C*-hexoside ^4^	0.445 ± 0.023 ^a^	0.368 ± 0.027 ^a^	0.357 ± 0.020 ^a,b^	0.347 ± 0.016 ^a,b^	0.250 ± 0.015 ^b,c^	0.146 ± 0.092 ^c^
Apigenin 8-*C*-hexoside 6-*C*-pentoside ^4^	3.05 ± 0.38 ^a^	2.45 ± 0.16 ^b^	2.35 ± 0.18 ^b^	2.42 ± 0.21 ^b^	1.68 ± 0.10 ^c^	1.41 ± 0.11 ^c^
Schaftoside	3.32 ± 0.10 ^a^	2.67 ± 0.18 ^b,c^	2.80 ± 0.034 ^b,c^	2.97 ± 0.28 ^a,b^	2.30 ± 0.23 ^c,d^	2.12 ± 0.18 ^d^
Isoorientin	14.3 ± 1.6 ^a^	11.3 ± 1.2 ^b^	11.8 ± 0.6 ^a,b^	12.8 ± 0.3 ^a,b^	11.3 ± 1.1 ^b^	7.90 ± 1.01 ^c^
Orientin	4.80 ± 0.30 ^a^	4.18 ± 0.41 ^a^	4.26 ± 0.22 ^a^	4.49 ± 0.09 ^a^	3.97 ± 0.43 ^a^	2.04 ± 0.30 ^b^
Vitexin	1.29 ± 0.06 ^a,b^	1.20 ± 0.12 ^ab^	1.30 ± 0.04 ^a,b^	1.33 ± 0.05 ^a^	1.09 ± 0.11 ^b^	0.684 ± 0.068 ^c^
DCG ^1^	0.172 ± 0.015 ^d^	0.470 ± 0.081 ^c^	0.397 ± 0.017 ^c,d^	0.577 ± 0.088 ^c^	0.790 ± 0.094 ^b^	1.35 ± 0.16 ^a^
Isovitexin	0.943 ± 0.092 ^a,b,c^	0.828 ± 0.076 ^b,c^	0.987 ± 0.033 ^a,b^	1.07 ± 0.02 ^a^	0.905 ± 0.068 ^a,b,c^	0.781 ± 0.067 ^c^
Caffeic acid derivative ^1^	1.06 ± 0.16 ^a^	0.558 ± 0.061 ^c^	0.854 ± 0.074 ^a,b^	1.10 ± 0.06 ^a^	0.878 ± 0.122 ^a,b^	0.741 ± 0.124 ^b,c^
Unknown ^1^	0.063 ± 0.004 ^c^	0.212 ± 0.009 ^a,b^	0.251 ± 0.009 ^a^	0.207 ± 0.018 ^a,b^	0.278 ± 0.067 ^a^	0.159 ± 0.016 ^b^
∑ Phenolic compounds	42.0 ± 3.5 ^a^	34.0 ± 2.7 ^b^	35. 1 ± 1.9 ^b^	38.0 ± 1.0 ^a,b^	32.7 ± 2.2 ^b^	24.9 ± 2.3 ^c^
∑ Flavone *C*-glycosides	36.8 ± 3.1 ^a^	30.3 ± 2.5 ^b^	31.0 ± 1.6 ^a,b^	33.0 ± 1.1 ^a,b^	27.6 ± 2.2 ^b^	21.0 ± 1.8 ^c^

^1^ Expressed as caffeic acid equivalent. ^2^ Expressed as orientin equivalent. ^3^ Expressed as *p*-coumaric acid equivalent. ^4^ Expressed as vitexin equivalent. Means with different letters in the same row are significantly different (*p* < 0.05).

**Table 4:** Contents of the individual phenolic compounds in the fresh matter (FM) of flax harvested at different growth stages (μg/g FM).

Compound	Stem Extension	Visible Bud	Flowering	Brown Capsule	Seed Ripening	Mature Seed
Coniferin ^1^	20.0 ± 0.5 ^b,c^	22.0 ± 4.9 ^b,c^	25.3 ± 3.2 ^b,c^	40.0 ± 7.8 ^b^	75.4 ± 15 ^a^	19.1 ± 3.2 ^c^
Caffeic acid hexoside I ^1^	19.6 ± 2.0 ^a^	12.5 ± 2.2 ^b,c^	18.1 ± 3.1 ^a,b^	16.4 ± 1.7 ^a,b^	12.8 ± 1.9 ^b,c^	7.45 ± 1.10 ^c^
Caffeic acid hexoside II ^1^	74.4 ± 7.4 ^a^	49.9 ± 3.2 ^b,c^	65.3 ± 12.8 ^a,b^	48.1 ± 3.2 ^b,c^	43.5 ± 8.2 ^c^	11.2 ± 3.1 ^d^
Lucenin-2 ^2^	45.4 ± 6.6 ^a^	45.1 ± 5.1 ^a^	47.9 ± 9.9 ^a^	42.7 ± 3.0 ^a^	38.5 ± 3.5 ^a,b^	24.1 ± 3.7 ^b^
*p*-Coumaric acid ethyl ester ^3^	6.96 ± 0.12 ^c,d^	8.48 ± 1.01 ^b,c^	10.6 ± 1.4 ^a^	9.76 ± 0.50 ^a,b^	9.49 ± 0.63 ^a,b^	5.93 ± 0.22 ^d^
Vicenin-2	107 ± 11 ^a,b^	102 ± 10 ^b^	132 ± 17 ^a^	107 ± 2 ^a,b^	89.9 ± 5.27 ^b,c^	48.3 ± 2.5 ^d^
Luteolin 6,8-*C*-hexoside-*C*-pentoside ^2^	117 ± 12 ^a,b^	117 ± 12 ^a,b^	146 ± 23 ^a^	133 ± 4 ^a^	135 ± 8 ^a^	84.4 ± 5.3 ^b^
Apigenin 6,8-di-*C*-hexoside ^4^	13.8 ± 0.9 ^a,b^	13.5 ± 1.4 ^a,b^	16.3 ± 2.3 ^a^	13.0 ± 0.4 ^a,b^	11.0 ± 0.7 ^b^	3.85 ± 2.37 ^c^
Apigenin 8-*C*-hexoside 6-*C*-pentoside ^4^	95.3 ± 15.0 ^a,b^	89.4 ± 8.8 ^a,b^	107 ± 17 ^a^	90.1 ± 5.6 ^a,b^	73.6 ± 2.1 ^b^	37.4 ± 2.1 ^c^
Schaftoside	104 ± 9 ^b^	97.3 ± 5.5 ^b^	128 ± 11 ^a^	111 ± 6 ^a,b^	101 ± 11 ^b^	56.3 ± 5.0 ^c^
Isoorientin	446 ± 64 ^a^	414 ± 50 ^a^	538 ± 73 ^a^	480 ± 26 ^a^	498 ± 60 ^a^	210 ± 23 ^b^
Orientin	150 ± 13 ^a^	152 ± 15 ^a^	194 ± 25 ^a^	168 ± 7 ^a^	174 ± 24 ^a^	54.0 ± 6.9 ^b^
Vitexin	40.1 ± 2.1 ^b^	43.7 ± 3.6 ^b^	59.3 ± 6.9 ^a^	49.7 ± 1.13 ^a,b^	47.8 ± 5.6 ^b^	18.2 ± 1.4 ^c^
DCG ^1^	5.35 ± 0.38 ^c^	17.3 ± 4.2 ^b^	18.0 ± 0.9 ^b^	21.7 ± 4.3 ^b^	34.7 ± 5.4 ^a^	35.7 ± 3.7 ^a^
Isovitexin	29.4 ± 3.3 ^b,c^	30.2 ± 3.4 ^b^	45.0 ± 5.3 ^a^	39.8 ± 1.9 ^a^	39.7 ± 4.0 ^a^	20.7 ± 1.3 ^c^
Caffeic acid derivative ^1^	33.1 ± 5.7 ^a,b^	20.4 ± 3.1 ^b^	39.1 ± 6.5 ^a^	41.0 ± 4.3 ^a^	38.6 ± 7.1 ^a^	19.7 ± 3.0 ^b^
Unknown ^1^	1.98 ± 0.24 ^d^	7.77± 0.84 ^b,c^	11.4 ± 0.8 ^a,b^	7.75 ± 1.09 ^b,c^	12.2 ± 3.1 ^a^	4.23 ± 0.36 ^c,d^
∑ Phenolic compounds	1309 ± 148 ^a^	1243 ± 123 ^a^	1601 ± 219 ^a^	1418 ± 62 ^a^	1436 ± 142 ^a^	660 ± 48 ^b^
∑ Flavone *C*-glycosides	1147 ± 133 ^a^	1105 ± 110 ^a^	1413 ± 190 ^a^	1233 ± 39 ^a^	1209 ± 123 ^a^	557 ± 37 ^b^

^1^ Expressed as caffeic acid equivalent. ^2^ Expressed as orientin equivalent. ^3^ Expressed as *p*-coumaric acid equivalent. ^4^ Expressed as vitexin equivalent. Means with different letters in the same row are significantly different (*p* < 0.05).

**Table 5:** Trolox equivalent antioxidant capacity (TEAC), ferric-reducing antioxidant power (FRAP), DPPH^•^ scavenging activity (expressed as EC_50_), and antioxidant activity in a β-carotene-linoleic acid emulsion system of flax harvested at different growth stages.

Growth Stage	TEAC		FRAP		EC_50_ (mg/mL)	Non-Oxidized β-Carotene (%) ^1^
	mmol TE/g Extract	µmol TE/g FM	mmol Fe^2+^/g Extract	µmol Fe^2+^/g FM		
Stem extension	0.184 ± 0.058 ^a^	5.78 ± 1.98 ^b,c^	0.763 ± 0.032 ^a,b^	23.8 ± 1.7 ^c^	0.167 ± 0.041 ^b,c^	68.6 ± 1.6 ^c,d^
Visible bud	0.150 ± 0.054 ^a^	5.46 ± 1.92 ^c^	0.715 ± 0.017 ^b,c^	26.1 ± 1.3 ^b,c^	0.201 ± 0.040 ^a,b^	68.0 ± 2.0 ^d^
Flowering	0.199 ± 0.011 ^a^	9.08 ± 1.23 ^a^	0.728 ± 0.024 ^a,b,c^	33.2 ± 3.6 ^a^	0.164 ± 0.015 ^b,c^	69.6 ± 2.2 ^c,d^
Brown capsule	0.222 ± 0.017 ^a^	8.11 ± 0.59 ^a,b^	0.783 ± 0.025 ^a^	28.7 ± 0.6 ^b^	0.152 ± 0.002 ^c^	73.7 ± 0.7 ^a,b^
Seed ripening	0.226 ± 0.022 ^a^	9.93 ± 1.39 ^a^	0.738 ± 0.028 ^a,b^	32.3 ± 1.9 ^a^	0.174 ± 0.014 ^b,c^	71.1 ± 1.4 ^b,c^
Mature seed	0.200 ± 0.018 ^a^	5.32 ± 0.39 ^c^	0.669 ± 0.060 ^c^	17.8 ± 1.3 ^d^	0.228 ± 0.014 ^a^	75.6 ± 1.9 ^a^

^1^ After 180 min of emulsion oxidation. FM, fresh matter; TE, Trolox equivalent; EC_50_, half-maximal effective concentration. Means with different letters in the same column are significantly different (*p* < 0.05).

## Data Availability

Data is contained within the article.
